# Supplementary material for: Vangl2 acts at the interface between actin and N-cadherin to modulate mammalian neuronal outgrowth
Source: eLife. 2020 Jan 7;9:e51822. doi: 10.7554/eLife.51822 (PMC6946565; doi:10.7554/eLife.51822)
Supplement: Transparent reporting form [file elife-51822-transrepform.docx]

***eLife’s* transparent reporting form**

We encourage authors to provide detailed information *within their submission* to facilitate the interpretation and replication of experiments. Authors can upload supporting documentation to indicate the use of appropriate reporting guidelines for health-related research (see [EQUATOR Network](http://www.equator-network.org/%20)), life science research (see the [BioSharing Information Resource](https://biosharing.org/)), or the [ARRIVE guidelines](http://www.plosbiology.org/article/info:doi/10.1371/journal.pbio.1000412) for reporting work involving animal research. Where applicable, authors should refer to any relevant reporting standards documents in this form.

If you have any questions, please consult our Journal Policies and/or contact us: [editorial@elifesciences.org](mailto:editorial@elifesciences.org).

**Sample-size estimation**

- You should state whether an appropriate sample size was computed when the study was being designed
- You should state the statistical method of sample size computation and any required assumptions
- If no explicit power analysis was used, you should describe how you decided what sample (replicate) size (number) to use

Please outline where this information can be found within the submission (e.g., sections or figure legends), or explain why this information doesn’t apply to your submission:

No statistical method was used to predetermine sample size. Animal experimental numbers corresponds to what is being used in the field and indicated in the legends. Care was taken to use the minimum number of animals that could reveal statistical significance using the indicated tests of significance.

**Replicates**

- You should report how often each experiment was performed
- You should include a definition of biological versus technical replication
- The data obtained should be provided and sufficient information should be provided to indicate the number of independent biological and/or technical replicates
- If you encountered any outliers, you should describe how these were handled
- Criteria for exclusion/inclusion of data should be clearly stated
- High-throughput sequence data should be uploaded before submission, with a private link for reviewers provided (these are available from both GEO and ArrayExpress)

Please outline where this information can be found within the submission (e.g., sections or figure legends), or explain why this information doesn’t apply to your submission:

The information about how often each experiment was performed can be found in the Quantification and Statistical Analysis section of the manuscript, page 18 line 753.

Independent experiments are considered as technical replications. Biological replications are defined by the total number of neurons analyzed per condition (control vs v*angl2* cKO).

Information about technical replication is given in the Quantification and Statistical Analysis section of the manuscript, page 18 line 753. For Biological replication information can be found in each figure legend, from page 20 to 24.

Handling of outliers can be found in the Quantification and Statistical Analysis section of the manuscript, page 18 lines 751-753.

No data were excluded

More details about the number of neurons analyzed for each experiment are available in figure legends.

**Statistical reporting**

- Statistical analysis methods should be described and justified
- Raw data should be presented in figures whenever informative to do so (typically when N per group is less than 10)
- For each experiment, you should identify the statistical tests used, exact values of N, definitions of center, methods of multiple test correction, and dispersion and precision measures (e.g., mean, median, SD, SEM, confidence intervals; and, for the major substantive results, a measure of effect size (e.g., Pearson's r, Cohen's d)
- Report exact p-values wherever possible alongside the summary statistics and 95% confidence intervals. These should be reported for all key questions and not only when the p-value is less than 0.05.

Please outline where this information can be found within the submission (e.g., sections or figure legends), or explain why this information doesn’t apply to your submission:

Information about statistical analysis methods can be found in the Quantification and Statistical Analysis section of the manuscript, page 18, lines 756-760.

Information about the statistical tests used can be found in the figure legends from page 20 to 24

P-values and confidence intervals can be found in the figure legends from page 20 to 24

(For large datasets, or papers with a very large number of statistical tests, you may upload a single table file with tests, Ns, etc., with reference to sections in the manuscript.)

**Group allocation**

- Indicate how samples were allocated into experimental groups (in the case of clinical studies, please specify allocation to treatment method); if randomization was used, please also state if restricted randomization was applied
- Indicate if masking was used during group allocation, data collection and/or data analysis

Please outline where this information can be found within the submission (e.g., sections or figure legends), or explain why this information doesn’t apply to your submission:

The experiments were not randomized but the scientist doing the recording was blind to the genotyping of the animals, and each experimental setup used littermates of both sex.

**Additional data files (“source data”)**

- We encourage you to upload relevant additional data files, such as numerical data that are represented as a graph in a figure, or as a summary table
- Where provided, these should be in the most useful format, and they can be uploaded as “Source data” files linked to a main figure or table
- Include model definition files including the full list of parameters used
- Include code used for data analysis (e.g., R, MatLab)
- Avoid stating that data files are “available upon request”

Please indicate the figures or tables for which source data files have been provided:

Examples of videos from which the data have been extracted are provided for: figure 3A-F; figure 4A-E; figure 4 F-G; figure 4-figure supplement 1E-K; figure 6 and figure 8.
